# Temporal Metabolic Reprogramming Reveals Stage-Specific Adaptations in Proso Millet Resistance Against Head Smut

**DOI:** 10.3390/metabo16040266

**Published:** 2026-04-16

**Authors:** Wenqi Fan, Mingyu Qi, Zhiguang Li, Yanyan Zuo, Min Zhao, Hanyu Liu, Yahui Wen, Xinxin Wang, Limei Bian, Liyuan Zhang

**Affiliations:** Chifeng Academy of Agricultural and Animal Husbandry Sciences, Chifeng 024031, China; fanwenqi@alu.cau.edu.cn (W.F.); qimingyu006@126.com (M.Q.); lizg05@126.com (Z.L.); yanyazuo2022@163.com (Y.Z.); zm0476@163.com (M.Z.); nyskyk@126.com (H.L.); 13947688085@163.com (Y.W.); wxx986@163.com (X.W.); bianlimei2013@126.com (L.B.)

**Keywords:** proso millet, *Sporisorium destruens*, metabolomics, phenylpropanoid biosynthesis

## Abstract

Background/Objectives: Proso millet (*Panicum miliaceum* L.), a drought-tolerant cereal vital to semi-arid agriculture, faces severe yield losses from head smut disease caused by the pathogen *Sporisorium destruens*. Although partial resistance exists, the dynamic molecular mechanisms governing its defense response across developmental stages remain poorly understood. Methods: Here, we performed untargeted metabolomics on leaf samples from Inoculated Asymptomatic (IA) and Inoculated Symptomatic (IS) plants of the partially resistant cultivar ‘Chishu 13’ at four key growth stages following pathogen inoculation, with group classification validated by qPCR. Using weighted metabolite co-expression network analysis (WGCNA) combined with differential metabolite screening, we identified 18 metabolites markedly enriched in the tricarboxylic acid (TCA) cycle, metabolite transport-related processes, and phenylpropanoid biosynthesis pathways. Results: Notably, L-phenylalanine accumulated substantially in IA plants relative to IS plants and correlated closely with biosynthesis of key defensive phenylpropanoids, including cinnamic acid and *p*-coumaric acid. Our results reveal distinct temporal patterns in metabolic reprogramming that correlate with resistance outcomes in Inoculated Asymptomatic plants: early stages are characterized by differential regulation of energy metabolism, while later stages show enhanced phenylpropanoid biosynthesis. These stage-specific metabolic adaptations are strongly associated with successful defense outcomes. Conclusions: These findings elucidate stage-specific metabolic adaptations that distinguish successful defense in IA plants from susceptibility in IS plants, providing robust biomarkers and stage-targeted strategies for breeding smut-resistant millet varieties.

## 1. Introduction

Proso millet (*Panicum miliaceum* L.), an important traditional minor cereal crop, possesses desirable agronomic traits such as drought tolerance, adaptation to poor soils, and a short growth cycle. However, its production is consistently constrained by the severe threat of head smut (*Sporisorium destruens*). As a typical systemic fungal disease, head smut is characterized by pathogen invasion at the seedling stage, followed by systemic colonization synchronized with host growth. Ultimately, it completely devastates the panicles by the heading stage, resulting in total yield loss [1,2,3]. Since no visible symptoms appear in the early stages and effective chemical control measures are lacking, the development of disease-resistant cultivars currently represents the most feasible control strategy [4,5]. In recent years, although partially resistant germplasm has been obtained through the screening of germplasm resources, there remains a lack of systematic understanding regarding the physiological and biochemical basis of disease resistance in proso millet, particularly the dynamic metabolic responses involved.

During pathogen infection, plants often undergo metabolic reprogramming to rapidly adjust their endogenous metabolic networks, thereby providing energy, constructing physical barriers, or synthesizing antimicrobial compounds [6,7]. Metabolomics enables the quantitative analysis of a broad spectrum of metabolites in organisms, allowing for the comparison of metabolic changes under different conditions and clarifying the associated physiological alterations and relevant metabolites [8,9]. In recent years, studies on related biotrophic pathogens have demonstrated that pathogenic fungi can actively interfere with host metabolic and developmental programs by secreting effector proteins. For example, Ustilago maydis (corn smut fungus) employs effectors to target host carbohydrate metabolism, hormone signaling, and secondary metabolic pathways to meet its nutritional demands while simultaneously suppressing plant immunity and inducing abnormal tissue differentiation [10,11]. Given that pathogens may disrupt normal host metabolic processes during infection, the observed metabolic differences between infected and healthy individuals could arise not only from the host’s active defense responses but also from metabolic dysregulation in susceptible materials caused by pathogen interference. In gramineous crops such as maize (*Zea mays* L.) and sorghum (*Sorghum bicolor* L.), head smut pathogens have been shown to interfere with host carbon and nitrogen metabolism, inhibit the allocation of photosynthetic products, and induce the activation of secondary metabolic pathways [12,13]. Correspondingly, individuals who successfully resist infection often exhibit earlier and stronger accumulation of phenylpropanoids, flavonoids, and volatile organic compounds. These metabolites not only possess direct antimicrobial activity but also function as signaling molecules involved in systemic acquired resistance [14,15]. However, current understanding of disease resistance-related metabolites in plants relies largely on indirect inferences from transcriptomic or proteomic data, lacking direct observation of metabolic dynamics throughout the entire pathogen infection process. In particular, no study has systematically delineated the evolution of metabolic profiles in proso millet from pathogen invasion to symptom outbreak. Although prior transcriptomic analyses have revealed significant differences in gene expression related to defense pathways such as phenylpropanoid and jasmonic acid (JA) biosynthesis between infected and healthy plants [16,17,18,19], it remains to be empirically demonstrated whether these transcriptional changes are truly translated into functional metabolic phenotypes and whether the temporal patterns of metabolic responses corresponding to different disease outcomes under the same genetic background exhibit fundamental differences.

Therefore, this study aims to systematically profile the dynamic metabolic changes in proso millet throughout *S. destruens* infection using untargeted metabolomics. By comparing infected and healthy plants across key growth stages under a uniform genetic background, we seek to identify resistance-related metabolites and pathways, thereby providing foundational insights for metabolic marker-assisted breeding.

## 2. Materials and Methods

### 2.1. Plant Materials

The proso millet variety ‘Chishu 13’, developed previously by our research group, was used in this study. Prior to sowing, seeds were artificially inoculated with head smut (*S. destruens*). The pathogen inoculum consisted of purified teliospore powder prepared from typical diseased panicles collected at the mature teliospore stage in the preceding year. The panicles were air-dried, ground, and sieved (200 mesh) to obtain the spore powder, which was then stored at 4 °C in darkness until use. Prior to inoculation, teliospore viability was confirmed: a spore suspension was plated on germination medium (1% agar + 1% sucrose), incubated at 24 °C in darkness for 24 h, and microscopically assessed for germ tube emergence. Only batches with ≥90% germination rate were used. Spore concentration was determined as 1.25 × 10^7^ teliospores per gram of powder using a hemocytometer. The dry mixing inoculation method was employed: teliospore powder and seeds were mixed at a mass ratio of 1:350, ensuring uniform coating of viable spores on seed surfaces.

Immediately after inoculation, seeds were broadcast-sown in plastic trays (30 cm × 20 cm) filled with sterilized field soil and placed in a controlled-environment growth chamber. Cultivation conditions were maintained at 28 °C, 80% relative humidity, 16 h photoperiod (light/dark), and a photosynthetically active radiation (PAR) of 600 μmol·m^−2^·s^−1^. Upon uniform seedling emergence, healthy seedlings were transplanted to an experimental field plot and established at a spacing of 20 cm between rows and 20 cm between plants under standard agronomic management. Throughout the entire growth period, plant growth status and disease development were continuously monitored and recorded. Concurrently, leaf samples were collected at key growth stages, rapidly frozen in liquid nitrogen, and subsequently stored at −80 °C for later metabolomic analysis. All plants in this study were uniformly inoculated with *S. destruens* teliospores; no non-inoculated control group was included. Plants were retrospectively categorized at the heading stage into two groups based on symptom development: the Inoculated Asymptomatic (IA) group (plants showing no visible disease symptoms throughout the experiment) and the Inoculated Symptomatic (IS) group (plants exhibiting typical head smut symptoms). This experimental design specifically isolates metabolic signatures associated with partial resistance under identical pathogen exposure conditions.

### 2.2. Sampling Strategy

A total of 200 individual proso millet plants were cultivated in this study. To comprehensively capture the metabolic dynamics during head smut infection, functional leaf samples were collected from all 200 plants at four key developmental stages: seedling, tillering, jointing, and heading. Immediately after collection, leaf samples were rapidly rinsed three times with ultrapure water on ice to remove surface contaminants and dust, gently blotted dry with lint-free absorbent paper, and then flash-frozen in liquid nitrogen to quench metabolic activity. Samples were subsequently labeled with plant ID and growth stage identifiers and stored at −80 °C. This process established an annotated longitudinal sample bank comprising 800 entries (200 plants × 4 time points). As the typical symptoms of head smut (black powdery sori on panicles) become fully apparent only at the heading stage, field phenotypes observed at this stage served as the final criterion for retrospective classification. Based on symptom development at the heading stage, the 200 inoculated plants were categorized into two groups: the Inoculated Symptomatic (IS) group (plants exhibiting typical head smut symptoms) and the Inoculated Asymptomatic (IA) group (plants showing no visible disease symptoms throughout the experiment). Subsequently, the corresponding preserved samples from all four preceding stages were retrospectively assigned to their respective groups for each plant. From each group, six representative individuals with stable and consistent phenotypes were randomly selected. Consequently, a total of 48 samples were subjected to metabolomic analysis, enabling a longitudinal comparison between the Inoculated Symptomatic and Inoculated Asymptomatic states (Table 1).

### 2.3. Quantification of Sporisorium destruens Biomass by qPCR

Fungal biomass was quantified by qPCR in the identical leaf samples used for metabolomics (seedling, tillering, jointing, and heading stages) following retrospective classification into Inoculated Symptomatic (IS) and Inoculated Asymptomatic (IA) groups. Genomic DNA was extracted (CTAB method), quantified (NanoDrop; A260/A280 ≈ 1.8–2.0), and verified by agarose gel electrophoresis. qPCR was performed on a Bio-Rad CFX96 system using *S. destruens*-specific *Ppi* primers and proso millet *GAPDH* as endogenous control (Table S1). Amplification specificity was confirmed by melt curve analysis and Sanger sequencing. Relative fungal biomass was calculated via the 2^−ΔΔCt^ method (normalized to *GAPDH*, referenced to IS at the heading stage). This analysis confirmed negligible pathogen load in IA plants across all stages, providing molecular validation for their use as the comparative reference group.

### 2.4. Metabolomic Analysis

Untargeted metabolomic profiling was performed using an ultra-high-performance liquid chromatography coupled with Fourier transform mass spectrometry (UHPLC-Orbitrap Exploris™ 240) system (Thermo Fisher Scientific, Waltham, MA, USA). The analysis was conducted by Shanghai Majorbio Bio Pharm Technology Co., Ltd. (Shanghai, China). Chromatographic separation was achieved on an HSS T3 column (100 mm × 2.1 mm, 1.8 μm). The mobile phases consisted of (A) water/acetonitrile (95:5, *v*/*v*) and (B) acetonitrile/isopropanol/water (47.5:47.5:5, *v*/*v*/*v*), both containing 0.1% formic acid. The flow rate was set at 0.40 mL/min, the column temperature was maintained at 40 °C, and the injection volume was 3 μL. Mass spectrometric detection was carried out in both positive and negative ionization modes with a mass scan range of *m*/*z* 70–1050. The ion source parameters were set as follows: spray voltages of +3500 V (positive) and −3000 V (negative); sheath gas flow, 50 arbitrary units (arb); auxiliary gas flow, 13 arb; ion source heater temperature, 450 °C; and collision energy stepped at 20, 40, and 60 V.

### 2.5. Data Analysis

Raw LC-MS/MS data from the 48 samples were processed using Progenesis QI (v 4.1) software (Waters Corporation, Milford, MA, USA) for peak detection, extraction, alignment, and integration. Metabolite annotation was performed against the HMDB, METLIN, and the in-house Majorbio database. To reduce technical variations introduced during experimentation and analysis, the annotated data were pre-processed as follows: First, metabolic features with more than 20% missing values in any sample group were removed. For the remaining features, missing values were imputed with half of the minimum positive value across all samples. The data were then normalized by total sum normalization to correct for variations in overall MS response intensity. Subsequently, variables showing a relative standard deviation (RSD) greater than 30% in the quality control (QC) samples were filtered out. Finally, the normalized data were subjected to a base-10 logarithmic (log_10_) transformation to generate the final data matrix for subsequent statistical analyses. Multivariate statistical analysis, including unsupervised principal component analysis (PCA), was performed using the ropls package (v1.6.2) in R. Functional annotation of differential metabolites was conducted based on the HMDB and KEGG pathway databases. Pathway enrichment analysis was carried out using scipy.stats module in Python (v 3.12).

## 3. Results

### 3.1. Consistent Phenotypic and Molecular Segregation in Response to S. destruens in ‘Chishu 13’

**Figure 1:**
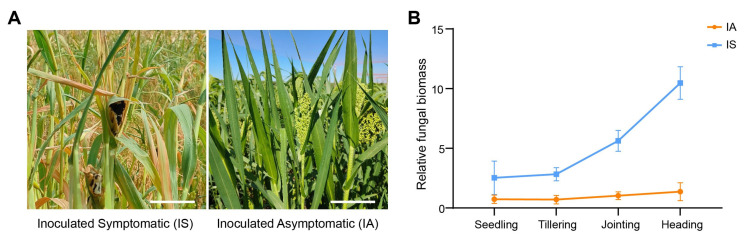
Characterization of the ‘Chishu 13’ response to head smut inoculation. (**A**) Phenotypic comparison between Inoculated Symptomatic (IS) plants (**left**) and Inoculated Asymptomatic (IA) plants (**right**) at the heading stage. Bar, 4 cm. (**B**) qPCR quantification of *S. destruens* biomass across four developmental stages.

Under artificial inoculation with *S. destruens*, the proso millet variety ‘Chishu 13’ consistently exhibited a stable partial resistance phenotype during three consecutive years (2023–2025) of field trials. The disease incidence rates were 47.0%, 50.5%, and 48.5% in 2023, 2024, and 2025, respectively, with a three-year average of 48.67% (Table 2), indicating high repeatability in its response to head smut. Phenotypic assessment at the heading stage enabled retrospective classification of all inoculated plants into two defined groups: the Inoculated Symptomatic (IS) group (exhibiting characteristic disease symptoms) and the Inoculated Asymptomatic (IA) group (remaining symptom-free throughout the experiment), as illustrated in Figure 1A. In the IS group, panicle development was severely impaired, with most failing to set grains; typical symptoms included replacement of panicles by digitate black teliosori composed of teliospores enveloped in a whitish hyphal membrane (Figure 1A, left). Conversely, IA plants maintained fully developed, grain-bearing panicles with no visible pathology (Figure 1A, right). Critically, qPCR quantification of fungal biomass across all developmental stages (Figure 1B) confirmed minimal to undetectable *S. destruens* levels in the IA group throughout the experiment, whereas the IS group exhibited progressive pathogen accumulation, with a marked increase initiating at the jointing stage. This molecular validation confirms that IA plants successfully restricted pathogen colonization despite uniform initial inoculation, thereby substantiating the biological rationale for using the IA group as the comparative reference.

### 3.2. Dynamic Metabolite Profiling in Response to S. destruens Colonization

**Figure 2:**
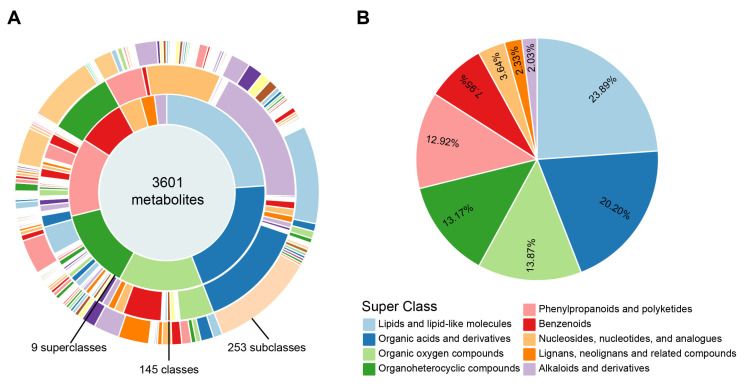
Metabolite classification and abundance in proso millet leaves from IA and IS plants. (**A**) Classification of 3601 annotated metabolites. (**B**) Relative abundance of major superclasses.

Based on the retrospectively classified Inoculated Symptomatic (IS) and Inoculated Asymptomatic (IA) groups defined in Section 3.1, leaf samples from IS and IA plants were collected at four key growth stages during the 2025 trial (*n* = 6 per group, 48 samples total) for untargeted metabolomic analysis (Table 1). A total of 3601 metabolites were detected, classified into 9 superclasses, 145 classes, and 253 subclasses (Figure 2A). At the superclass level, Lipids and lipid-like molecules constituted the highest proportion (23.89%), followed by Organic acids and derivatives (20.20%) and Organic oxygen compounds (13.87%) (Figure 2B), indicating that lipid and organic acid metabolites dominate the proso millet leaf metabolome.

**Figure 3:**
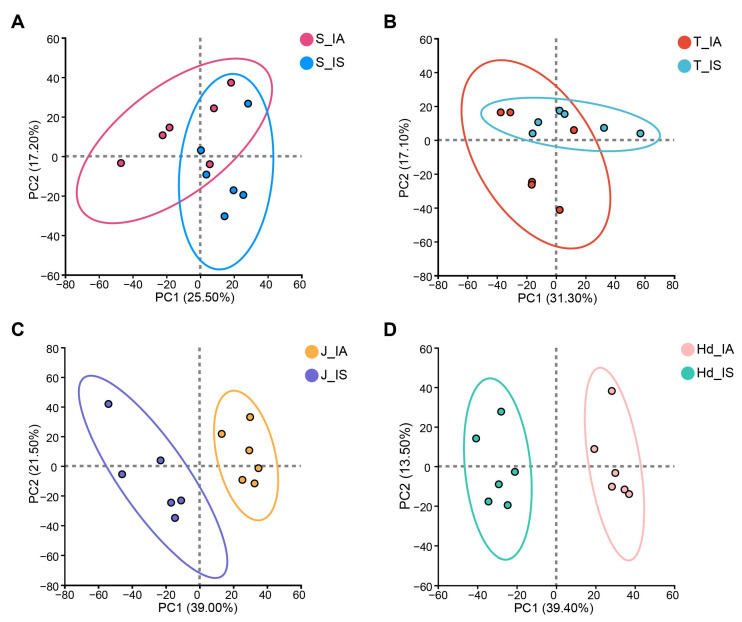
Principal component analysis of metabolite profiles from IA and IS proso millet across four developmental stages. (**A**–**D**) Overlap at early stages, progressive separation along PC1 from jointing to heading, with maximum divergence at heading.

To dissect the dynamic metabolic impact of differential pathogen outcomes, PCA was performed per growth stage. Significant metabolic divergence emerged between the IS and IA groups across development. At the seedling and tillering stages, IS and IA samples showed high overlap in PCA score plots (Figure 3A,B), indicating negligible metabolic differentiation during early infection. Beginning at jointing, clear separation along PC1 emerged, intensifying markedly at the heading stage (Figure 3C,D). This trajectory demonstrates that successful pathogen colonization in the IS group—but not in the IA group—triggers substantial, stage-dependent metabolic reprogramming during mid-to-late development.

### 3.3. Metabolite and Pathway Alterations at Jointing and Heading Stages

**Figure 4:**
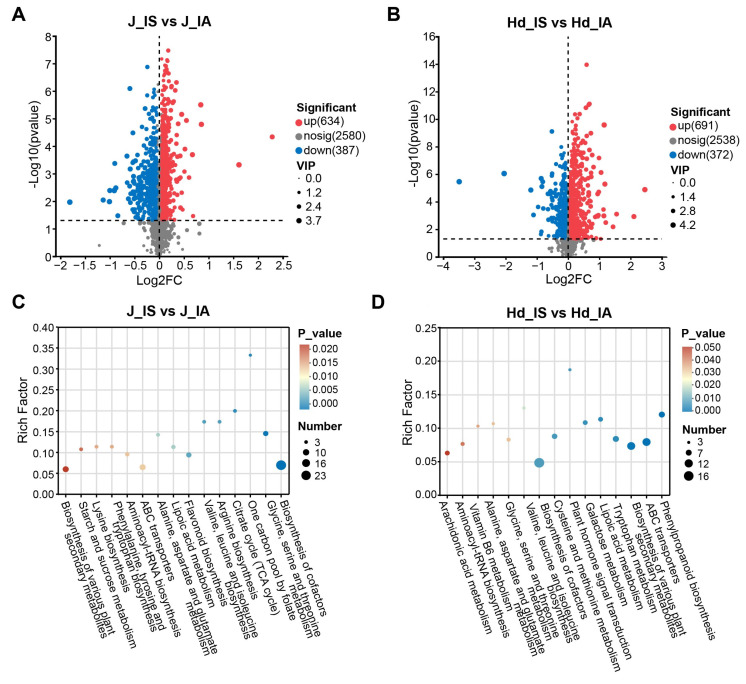
Metabolite and pathway dynamics between IA and IS groups at the jointing and heading stages. (**A**,**B**) Volcano plots of DEMs at jointing and heading stages. (**C**,**D**) KEGG enrichment analysis of DEMs at jointing and heading stages.

To identify the key metabolic features underlying the response of ‘Chishu 13’ to head smut infection, we focused on comparing the metabolic differences between infected and healthy plants at the jointing and heading stages, both of which showed clear metabolomic separation in the PCA score plots (Figure 3C,D). Applying strict screening criteria (fold change ≥ 1, OPLS-DA VIP ≥ 1, *p* < 0.05), a total of 1021 significantly differential metabolites (DEMs) were identified at the jointing stage, of which 634 were up-regulated and 387 were down-regulated (Figure 4A). At the heading stage, 1063 DEMs were detected (691 up-regulated and 372 down-regulated) (Figure 4B). Notably, both the number of DEMs and the magnitude of their changes increased substantially from the jointing to the heading stage, consistent with the progressively severe disease symptoms observed during later growth phases.

KEGG pathway enrichment analysis of the DEMs revealed that several pathways related to energy metabolism and plant defense were significantly enriched at different stages. At the jointing stage, the significantly enriched pathways are primarily involved in energy and basic metabolic processes, such as the citrate cycle (TCA cycle), biosynthesis of cofactors, and certain amino acid metabolism pathways, suggesting that the metabolic perturbation at this stage may be associated with adjustments in cellular energy status and redox balance (Figure 4C). In contrast, the enriched pathways at the heading stage shifted notably toward plant-specific metabolism and transport processes, including phenylpropanoid biosynthesis, biosynthesis of various plant secondary metabolites, metabolites associated with ABC transporter substrates, and plant hormone metabolism (Figure 4D). These results indicate that disturbances related to basic metabolism may already have begun at the jointing stage, while key pathways involved in secondary metabolism were significantly activated at the heading stage. The accumulation of corresponding metabolites may partly reflect plant stress responses or tissue damage processes during the later phases of infection.

### 3.4. Candidate Resistance Metabolites Identified by Integrated Screening

**Figure 5:**
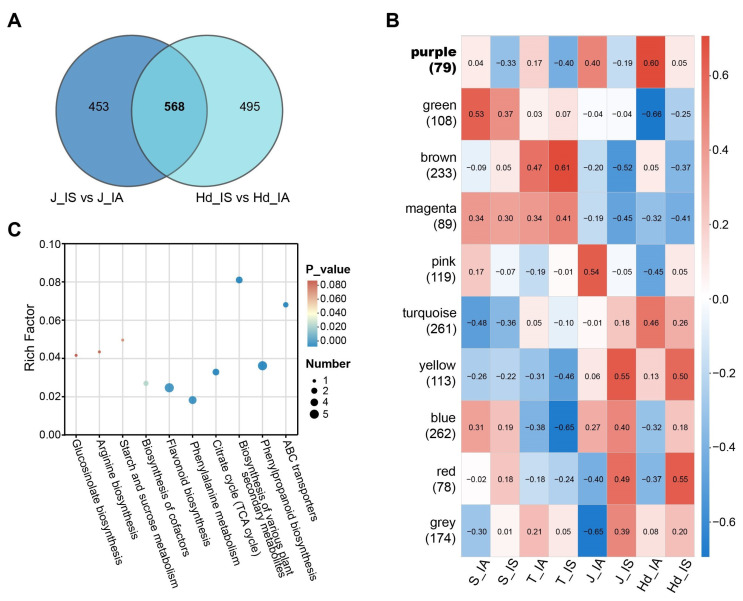
Identification of key candidate metabolites through integrated metabolomic analyses comparing IA and IS plants. (**A**) Venn diagram of DEMs shared between jointing and heading stages (n = 568). (**B**) Module-trait correlation plot from WGCNA. (**C**) KEGG pathway enrichment of metabolites in the purple module.

To further elucidate the relationship between the stable head smut-resistant phenotype and the metabolic regulatory network in proso millet, we performed dual analyses on all differential metabolites identified at the jointing and heading stages (1516 in total). First, intersection analysis yielded 568 common DEMs that were significantly altered at both developmental stages (Figure 5A). Second, weighted metabolite co-expression network analysis (WGCNA) conducted on all 1516 DEMs revealed that the purple module (containing 79 metabolites) showed the highest positive correlation with the healthy phenotype and a significant negative correlation with the infected phenotype (Figure 5B), suggesting its potential involvement in regulating resistance responses; thus, this module was designated as the target module.

Subsequently, to screen for key candidate metabolites, we intersected the purple module with the 568 common DEMs, ultimately obtaining 18 high-confidence candidate metabolites (Table S1). Functional annotation indicated that metabolites within the purple module were primarily enriched in pathways such as phenylpropanoid biosynthesis, phenylalanine metabolism, the TCA cycle, and metabolites potentially transported by ABC transporters (Figure 5C). These pathways collectively cover several key biological processes, including energy metabolism, biosynthesis of defensive compounds, and transport.

### 3.5. Temporal Dynamics and Functional Characterization of Key Metabolites Distinguishing IA and IS Phenotypes

**Figure 6:**
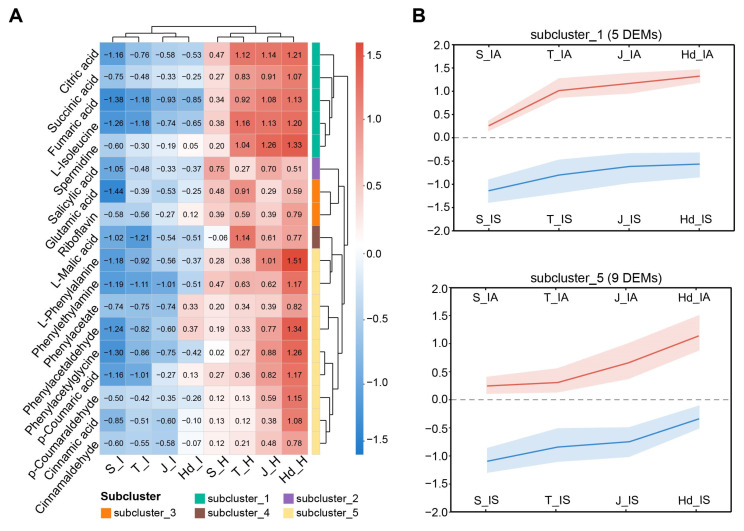
Temporal dynamics of key metabolites distinguishing IA and IS plants. (**A**) Clustering of 18 DEMs separates IA and IS samples. (**B**) Subcluster 1 accumulates from tillering, and Subcluster 5 rises from jointing.

Metabolite–phenotype correlation analysis demonstrated that the 18 candidate DEMs effectively distinguished IA from IS phenotypes, with samples clearly clustering according to disease outcome (Figure 6A). Hierarchical clustering further divided these metabolites into five subclusters (Subclusters 1–5) exhibiting distinct temporal accumulation patterns that differentially correlate with resistance phenotypes. Among them, Subcluster 1 (5 metabolites) and Subcluster 5 (9 metabolites) represented the two most prominent subclusters.

Metabolites in Subcluster 1 showed distinct accumulation patterns in IA versus IS plants starting from the tillering stage (Figure 6B). KEGG pathway enrichment analysis indicated that these metabolites were primarily involved in the TCA cycle and metabolites associated with cellular transport processes (Table S2). Key intermediates such as citrate and isocitrate exhibited progressively higher abundance in IA plants compared to IS plants throughout development. While these metabolic differences correlate strongly with the resistant phenotype, their functional role in defense requires further validation, as they may reflect either host adaptive responses or pathogen-induced metabolic reprogramming. In contrast, metabolites in Subcluster 5 began to accumulate significantly in the IA plants at the jointing stage and remained at high levels during the heading stage (Figure 6B). Enrichment analysis revealed that this subcluster was significantly associated with phenylalanine metabolism and phenylpropanoid biosynthesis. Representative metabolites included phenylalanine derivatives (phenylacetic acid, phenylethylamine) and phenylpropanoid precursors (cinnamic acid, *p*-coumaric acid), suggesting that this pathway plays a key role in the secondary metabolic response of proso millet against head smut (Table S1).

**Figure 7:**
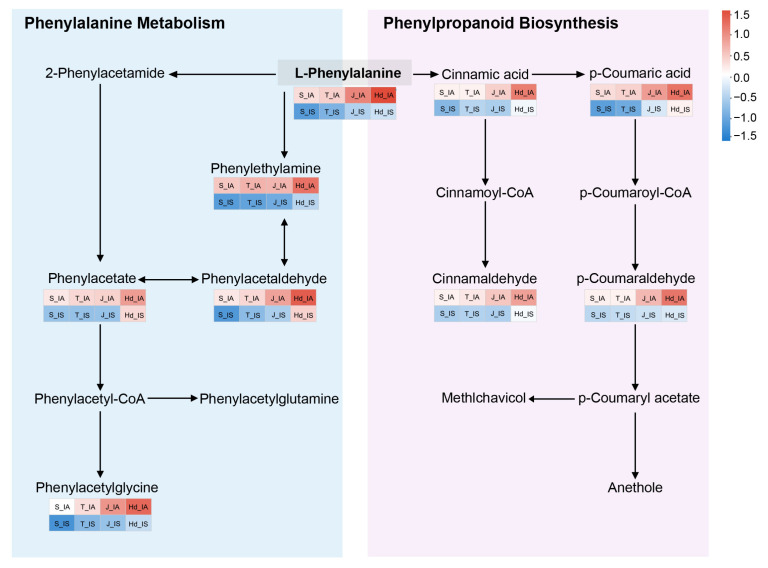
Metabolic pathway map of Subcluster 5 metabolites identified from IA versus IS comparisons.

To further elucidate the metabolic network features of this subcluster, we mapped the metabolic pathways involved in Subcluster 5 (Figure 7). The results showed that L-phenylalanine, as a central node metabolite in this network, consistently maintained high abundance in the healthy group but significantly decreased in the infected group. The difference between the two groups was most pronounced at the heading stage, indicating that L-phenylalanine likely serves as a hub in regulating disease-resistant metabolism during the later phases of infection.

## 4. Discussion

Our experimental design using Inoculated Asymptomatic (IA) plants as the reference group rather than non-inoculated controls provides unique insights into partial resistance mechanisms. This approach specifically isolates metabolic differences associated with successful defense against *S. destruens* after pathogen exposure, eliminating confounding variables related to differential initial exposure. The near-complete absence of pathogen biomass in the Inoculated Asymptomatic group (Figure 1B) validates this comparative approach for identifying true resistance-associated metabolic signatures.

Head smut is a key fungal disease threatening the safe production of proso millet, severely affecting grain yield and quality [1,3]. Although previous studies have identified several disease resistance-related genes at the genetic and transcriptional levels [14], the dynamic metabolic regulatory mechanisms underlying the response of proso millet to *S. destruens* infection remain unclear. To address this, the present study employed time-series metabolomics to analyze the metabolic profiles of both Inoculated Asymptomatic (IA) and Inoculated Symptomatic (IS) plants of the proso millet variety ‘Chishu 13’ across the entire growth period. It should be noted that the selection of a single partially resistant cultivar was intentional to minimize genetic background variation and precisely capture temporal metabolic dynamics; validation of the identified metabolic signatures across diverse proso millet germplasm with graded resistance levels is recognized as an essential next step for confirming biomarker universality. This approach systematically revealed the dynamic metabolic reprogramming and its temporal characteristics in proso millet during head smut infection.

During the early infection stages (seedling and tillering), the metabolic profiles of IS and IA sample plants did not exhibit significant separation (Figure 3A,B), indicating that during initial pathogen colonization, the host had not yet activated specific metabolic responses detectable by metabolomics. Alternatively, the physiological changes at this phase might primarily occur at the molecular signaling level and have not yet been systematically reflected in the overall metabolic network. However, a critical transition in the metabolic phenotype occurred beginning at the jointing stage. Principal component analysis (PCA) showed that the two groups of samples diverged distinctly along PC1, reaching maximal separation at the heading stage (Figure 3C,D). This temporal dynamic closely aligns with the latent and progressive nature of head smut, suggesting that the host’s metabolic regulatory capacity prior to systemic pathogen spread is a key determinant of the ultimate resistant or susceptible phenotype.

Further metabolic pathway analysis revealed that the differential metabolites at the jointing stage were significantly enriched in TCA cycle and cofactor metabolism pathways (Figure 4C). Notably, TCA cycle intermediates such as citrate and isocitrate showed higher relative abundance in IA plants compared to IS plants (Figure 6B), suggesting differential metabolic regulation between phenotypic groups. The differential accumulation of TCA cycle intermediates could reflect either IA plants’ capacity to maintain metabolic homeostasis under pathogen challenge or IS plants’ metabolic disruption due to successful colonization. While these metabolites provide carbon skeletons for potential defense compound synthesis, their specific contribution to resistance mechanisms requires functional validation [20,21,22]. Furthermore, the enrichment of metabolites related to cellular transport systems indicates that the cellular membrane transport system may participate in maintaining intracellular homeostasis through the trafficking of defense compounds [23,24,25,26]. It should be noted that our metabolomic analysis detected substrates and products associated with transport processes rather than direct measurement of transporter activity, expression, or protein function. Future transcriptomic, proteomic, or functional validation studies will be necessary to confirm the involvement of specific ABC transporters in the resistance response. While the observed metabolic correlations strongly imply pathway-level shifts, future integration of enzyme activity assays and multi-omics data will further elucidate the regulatory mechanisms underlying these changes.

By the heading stage, metabolic divergence was further intensified. The phenylalanine metabolism and phenylpropanoid biosynthesis pathways emerged as the most significantly enriched differential pathways (Figure 4D). Additionally, metabolites associated with phytohormone biosynthesis and metabolism were differentially accumulated, suggesting potential alterations in hormonal status. Weighted metabolite co-expression network analysis (WGCNA) further corroborated these findings, revealing that metabolites within the purple module—including L-phenylalanine, cinnamic acid, *p*-coumaric acid, phenylacetic acid, and phenethylamine—were highly accumulated in IA plants and occupied central positions in the metabolic network (Figure 5B. As a pivotal hub molecule in plant defense metabolism, phenylalanine significantly influences the strength of disease resistance (Figure 7). Its downstream products, cinnamic acid and *p*-coumaric acid, serve dual roles: they act not only as precursors for lignin synthesis (contributing to cell wall thickening to physically block pathogen invasion) but also exhibit direct antifungal activity [27,28,29,30,31,32]. Furthermore, the metabolic crosstalk between this pathway and salicylic acid (SA)-mediated systemic acquired resistance (SAR) suggests the existence of multi-layered synergistic defense mechanisms [33,34,35]. In contrast, the metabolic flux through this pathway was significantly down-regulated in IS plants, resulting in the failure to effectively activate defense responses.

**Figure 8:**
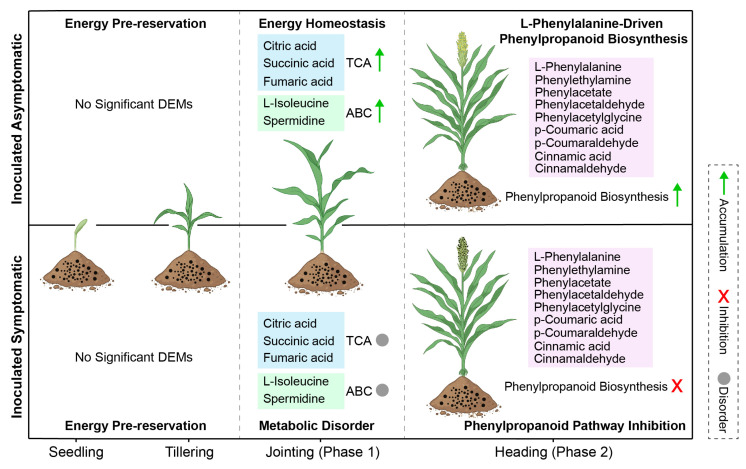
Schematic representation of the two-phase metabolic response model in IA versus IS proso millet during *S. destruens* challenge. The schematic was created in BioRender. Wenqi, F. (2026) https://BioRender.com/qug4teh.

Figure 8 presents a conceptual model of stage-specific metabolic adaptations in proso millet resistance against *S. destruens*, based on correlative metabolomic evidence. This model delineates two temporally distinct metabolic phases that strongly correlate with resistance outcomes. During early infection (seedling to tillering stages), IA and IS plants exhibit no discernible metabolic divergence (Figure 3A,B), suggesting that initial pathogen exposure triggers similar metabolic responses regardless of ultimate disease outcome. A critical transition occurs at the jointing stage, where IA plants—but not IS plants—maintain a higher abundance of TCA cycle intermediates and metabolites associated with cellular transport processes (Figure 6B). This metabolic distinction precedes visible symptom development and strongly correlates with subsequent disease outcomes, though causal relationships require functional validation. By the heading stage, a second distinct metabolic phase emerges in IA plants, characterized by pronounced enrichment of phenylalanine pathway metabolites (L-phenylalanine, cinnamic acid, *p*-coumaric acid) and enhanced phenylpropanoid biosynthesis. While these metabolites are known to contribute to defense through cell wall reinforcement and antimicrobial activity in other systems [27,28,29,30,31,32], their specific functional contribution to resistance in proso millet requires direct experimental validation. Importantly, the sequential emergence of these two metabolic phases—first energy homeostasis followed by defense compound synthesis—suggests a coordinated temporal progression rather than isolated responses, though this coordination hypothesis remains to be functionally tested. While our data reveal significant shifts in metabolites related to hormone metabolism and cellular transport, direct evidence of altered signaling or transporter activity would require integration with proteomic and transcriptomic analyses in future studies. In contrast, IS plants fail to activate this pathway effectively due to compromised metabolic flux, resulting in defense collapse. We emphasize that this model represents a hypothesis derived from correlative evidence. While the temporal sequence and strong association with resistance phenotypes suggest functional relevance, causation cannot be established from metabolomic data alone. Functional validation through exogenous metabolite supplementation, targeted genetic manipulation, or enzyme activity assays will be essential to determine whether these metabolic patterns constitute an active defense strategy or are secondary consequences of resistance mechanisms.

Based on this temporal regulatory framework, we identified 18 key DEMs (Table S2), spanning TCA cycle intermediates, phenylalanine derivatives, and characteristic heterocyclic compounds. These metabolites not only provide mechanistic insights into the stage-specific metabolic reprogramming underlying proso millet resistance to head smut but also represent promising biomarker candidates for early resistance prediction. Their validation may facilitate precision screening of resistant germplasm and inform metabolic engineering strategies for sustainable disease management in millet breeding programs.

## 5. Conclusions

In summary, this study establishes a temporal metabolic framework strongly associated with the partial resistance phenotype in proso millet. We identify two sequentially emerging metabolic phases in Inoculated Asymptomatic plants that correlate with successful defense: an early phase characterized by differential regulation of energy metabolism (jointing stage), followed by a late phase featuring enhanced phenylpropanoid biosynthesis (heading stage). While these stage-specific metabolic patterns are robustly associated with resistance outcomes, establishing their causal role in defense requires functional validation. The identification of stage-specific metabolic signatures distinguishing IA from IS plants—particularly L-phenylalanine as a pivotal regulatory node in the phenylpropanoid pathway—provides testable hypotheses for mechanistic studies and actionable biomarkers for breeding. Critically, this coordinated response is absent in Inoculated Symptomatic (IS) plants, which exhibit metabolic dysregulation and progressive pathogen accumulation. These findings underscore the importance of dynamic metabolic perspectives in plant–pathogen interactions and offer a foundation for developing metabolite-guided strategies to enhance disease resilience in millet and related cereals.

## Figures and Tables

**Table 1:** Sampling information for metabolite analysis.

Sampling Stage	Phenotypic Grouping	Number of Samples	Abbreviation
Seedling emergence	Inoculated Asymptomatic	6	S_IA
	Inoculated Symptomatic	6	S_IS
Tillering	Inoculated Asymptomatic	6	T_IA
	I Inoculated Symptomatic	6	T_IS
Jointing	Inoculated Asymptomatic	6	J_IA
	Inoculated Symptomatic	6	J_IS
Heading	Inoculated Asymptomatic	6	Hd_IA
	Inoculated Symptomatic	6	Hd_IS

**Table 2:** Statistics of disease incidence in *S. destruens*-inoculated ‘Chishu 13’ plants.

Year	Number of Inoculated Plants	Number of IS Plants	IS Rate (%)
2023	200	94	47.0
2024	200	101	50.5
2025	200	97	48.5

## Data Availability

The original contributions presented in this study are included in the article and Supplementary Material. Further inquiries can be directed to the corresponding author.

## Supplementary Materials

The following supporting information can be downloaded at https://www.mdpi.com/article/10.3390/metabo16040266/s1, Table S1. qPCR primer sequences. Table S2. Annotation information of the 18 DEMs.
